# ^99m^Tc-HYNIC-IL-2 scintigraphy to detect acute rejection in lung transplantation patients: a proof-of-concept study

**DOI:** 10.1186/s13550-019-0511-z

**Published:** 2019-05-10

**Authors:** Eef D. Telenga, Wim van der Bij, Erik F. J. de Vries, Erik A. M. Verschuuren, Wim Timens, Gert Luurtsema, Riemer H. J. A. Slart, Alberto Signore, Andor W. J. M. Glaudemans

**Affiliations:** 1 Medical Imaging Center, Department of Nuclear Medicine and Molecular Imaging, University of Groningen, University Medical Center Groningen, Hanzeplein 1, 9700 RB Groningen, The Netherlands; 2 Department of Respiratory Diseases, University of Groningen, University Medical Center Groningen, Groningen, The Netherlands; 3 Department of Pathology, University of Groningen, University Medical Center Groningen, Groningen, The Netherlands; 4 Department of Biomedical Photonic Imaging, University of Twente, Enschede, The Netherlands; 5 Nuclear Medicine Unit, Department of Medical-Surgical Sciences and Translational Medicine, Sapienza University of Rome, Rome, Italy

**Keywords:** ^99m^Tc-HYNIC-IL-2 scintigraphy, Lung transplantation, Rejection, SPECT/CT, Imaging

## Abstract

**Rationale:**

Acute allograft rejection is one of the major complications after lung transplantation, and adequate and early recognition is important. Till now, the reference standard to detect acute rejection is the histopathological grading of transbronchial biopsies (TBBs). Acute rejection is characterised by high levels of activated T lymphocytes. Interleukin-2 (IL-2) binds specifically to high-affinity IL-2 receptors expressed on the cell membrane of activated T lymphocytes. The aim of this proof-of-concept study was to evaluate if non-invasive imaging with ^99m^Tc-HYNIC-IL-2 is able to detect acute rejection after lung transplantation.

**Methods:**

^99m^Tc-HYNIC-IL-2 scintigraphy (static, SPECT/CT of the lungs) was performed shortly before routine transbronchial biopsy (pathology as reference standard). Scans were scored as likely or unlikely for rejection, and semiquantitative analysis (target-to-background ratio) was performed.

**Results:**

Thirteen patients were included of which 3 showed acute rejection at transbronchial biopsy; in 2 of these patients (scored as graded 2–3 at pathology), the scan was scored likely for rejection, and in 1 patient (scored grade 1 at pathology), the scan was scored unlikely. No correlation was found between biopsy results and semiquantitative analysis.

**Conclusion:**

^99m^Tc-HYNIC-IL-2 scintigraphy proved to be a good technique to detect grade 2 and 3 acute rejection in a small sample population of patients after lung transplantation. Larger studies are necessary to really show the added value of this non-invasive specific imaging technique over transbronchial biopsy. Alternatively, imaging with the PET tracer ^18^F-IL-2 may be useful for this purpose.

## Background

Lung transplantation is a therapeutic option in patients with end-stage pulmonary disease. Acute allograft rejection is one of the major complications after transplantation, and adequate and early recognition is of invaluable importance. However, diagnosing and monitoring acute rejection can be difficult. Clinical signs are non-specific and are not able to differentiate between rejection and other causes of graft dysfunction. Acute rejection may initially even be clinically silent [1]. High-resolution computed tomography (HR-CT) has an accuracy of only 53% (with sensitivity 35% and specificity 73%) for the diagnosis of acute rejection, and no individual HR-CT finding is significantly correlated with this diagnosis [2]. Other biomarkers such as C-reactive protein (CRP) and erythrocyte sedation rate (ESR) are insensitive and non-specific. More recently, the measurement of several cytokines in bronchoalveolar lavage samples has been proposed [3]. The reference standard to detect acute rejection is still the histopathological grading of transbronchial biopsies (TBBs). To date, in absence of a more specific non-invasive tool for the diagnosis of acute rejection, many centres perform surveillance TBB at fixed intervals during the first postoperative year, in addition to TBB in case of new symptoms or signs of rejection [4]. However, TBB has several limitations; it may cause bleeding and complications and may lead to sample errors. Therefore, a non-invasive imaging tool to detect rejection after transplantation is certainly needed.

Histopathological lesions, observed in acute rejection, show perivascular and interstitial mononuclear cell infiltrates in the pulmonary allograft [5], which are characterised by high levels of activated cytotoxic T lymphocytes overexpressing high-affinity interleukin-2 receptors (IL-2R), thus rendering T cells highly responsive to interleukin-2 (IL-2) [1]. IL-2 binds specifically to high-affinity IL-2R expressed on the cell membrane of activated T lymphocytes [6]. When labelled with a suitable radionuclide, IL-2 could be used as a probe to visualise lymphocyte infiltration by nuclear molecular imaging. To date, clinical research with radiolabelled IL-2 (e.g. ^99m^Tc-HYNIC-IL-2 [7]) has been performed in various inflammatory diseases in over 1000 patients (inflammatory bowel disease, atherosclerosis, thyroiditis, diabetes type 1, etc.) [6, 8, 9].

In this proof-of-concept study, we investigated if acute allograft rejection can be detected by ^99m^Tc-HYNIC-IL-2 scintigraphy, including single-photon emission computed tomography/computed tomography (SPECT/CT), in lung transplant recipients shortly after transplantation.

## Methods and materials

Thirteen lung transplant recipients were included in this study. All recipients were > 18 years and provided written informed consent. Maintenance immunosuppression consisted of tacrolimus, mycophenolate mofetil, and prednisolone. Patients were clinically assessed prior to the scan to see if they had ongoing viral infections. The study was approved by the local Medical Ethics Committee (trial number 2009/219).

^99m^Tc-HYNIC-IL-2 scintigraphy was performed shortly before the first routine bronchoscopy (TBB as reference standard) after transplantation (median 1 day, maximum 15 days). This bronchoscopy was performed several weeks after transplantation (median 36 days, minimum 19 days, maximum 126 days). ^99m^Tc-HYNIC-IL-2 was produced as previously described [7], and the preferred injection dose was 185 MBq (range of administered dose 92–192 MBq). Planar anteroposterior images of the thorax and abdomen were acquired 60 min (range 55–67) after the administration of the radiopharmaceutical, followed by SPECT/CT of the thorax, SPECT for quantification, and low-dose CT for anatomic co-localization and attenuation correction. All images were acquired on a SPECT/CT gamma camera system (Siemens Symbia T, Siemens Medical Systems, Knoxville, TN, USA). The scans were analysed by two experienced nuclear medicine specialists (AG, RS) who were blinded to all patient information except sex, type of transplant (unilateral or bilateral), and time between transplantation and scan. The reviewers assessed the scans and scored the likelihood of acute rejection (rejection unlikely or rejection likely). If there was a disagreement between the reviewers, the case was reviewed together for consensus. The planar images were scored according to a grading system: grade 0, no uptake; grade 1, uptake lower than mediastinum; grade 2, uptake equal to mediastinum; and grade 3, uptake higher than mediastinum. Grades 2 and 3 were scores as rejection likely, and grades 0 and 1 as rejection unlikely. For the SPECT/CT images, the grading system was the same, but the grading scores were given for each lobe. If at least one lobe was scored with grade 2 or 3, this was regarded as rejection likely. Semiquantitative analysis on SPECT/CT images was performed by drawing the volume of interests around the lungs and dividing the number of counts per millilitre in the lung(s) by the counts per millilitre in reference tissues (aorta, bone marrow, and muscle) to calculate the target-to-background ratio (T/B). TBBs were assessed by an experienced pulmonary pathologist (WT). The TBBs were graded according to the criteria of the International Society for Heart and Lung Transplantation (ISHLT) [10]. On histology, acute rejection was defined as an ISHLT A score ≥ 1.

Correlation between IL-2 scintigraphy results and histology was assessed with Spearman’s rho. Additionally, sensitivity, specificity, positive predictive value (PPV), and negative predictive value (NPV) with 95% confidence intervals (95% CI) were calculated.

## Results

In Table 1 the characteristics of the 13 patients are presented. None of the patients showed signs of viral infection. Ten patients underwent bilateral lung transplantation and 3 unilateral transplantation. In 2 patients, no TBBs were available. In 1 patient, there was a non-accessible stenosis of the airway anastomosis during bronchoscopy. In the other patient, no assessable material was found in the biopsy. Three patients showed acute rejection in the TBB. In 2 of these patients, the scan was scored (both on planar as on SPECT images) as rejection likely. In the other patient, the scan was scored as rejection unlikely. In patients without rejection in the biopsies, no scans were scored as rejection likely. The correlation between the rejection on biopsies and the visual assessment of the scans was 0.77 (*p* = 0.006). The calculated sensitivity was 67% (95% CI 13–100%), the specificity was 100%, the positive predictive value was 100%, and the negative predictive value was 89% (68–100%). No correlation was found between the rejection on biopsies and the T/B ratios on SPECT/CT (data not shown).

**Table 1:** Patient characteristics

Pt	Age (years)	Sex	Indication for transplant	Type of transplant	Pathology					IL-2 imaging
					ISHLT A	ISHLT B	ISHLT C	ISHLT D	Assessment of acute rejection	
1	46	Female	Pulmonal hypertension	Bilateral	0	0	0	0	Rejection unlikely	Rejection unlikely
2	47	Female	COPD	Unilateral (L)	0	0	0	0	Rejection unlikely	Rejection unlikely
3	30	Male	Cystic fibrosis	Bilateral	–	1R	0	0	–	Rejection unlikely
4	51	Male	COPD	Bilateral	2	0	1	0	*Rejection*	*Rejection likely*
5	48	Female	Pulmonal hypertension	Bilateral	0	0	0	0	Rejection unlikely	Rejection unlikely
6	52	Female	COPD	Bilateral	0	1R	0	0	Rejection unlikely	Rejection unlikely
7	63	Male	COPD	Bilateral	0	0	0	0	Rejection unlikely	Rejection unlikely
8	62	Male	Fibrosis	Bilateral	–	–	–	–	–	Rejection unlikely
9	53	Male	COPD	Bilateral	0	1R	0	0	Rejection unlikely	Rejection unlikely
10	38	Male	Cystic fibrosis	Bilateral	0	0	0	0	Rejection unlikely	Rejection unlikely
11	59	Female	COPD	Unilateral (R)	0	0	0	0	Rejection unlikely	Rejection unlikely
12	47	Male	Alpha-1-antitrypsine deficiency	Bilateral	3	1R	0	0	*Rejection*	*Rejection likely*
13	64	Female	Bronchiolitis	Unilateral (R)	1	0	0	0	*Rejection*	Rejection unlikely

*COPD* chronic obstructive pulmonary disease, *ISHLT* The International Society for Heart and Lung Transplantation, *IL-2* interleukin-2

**Fig. 1:**
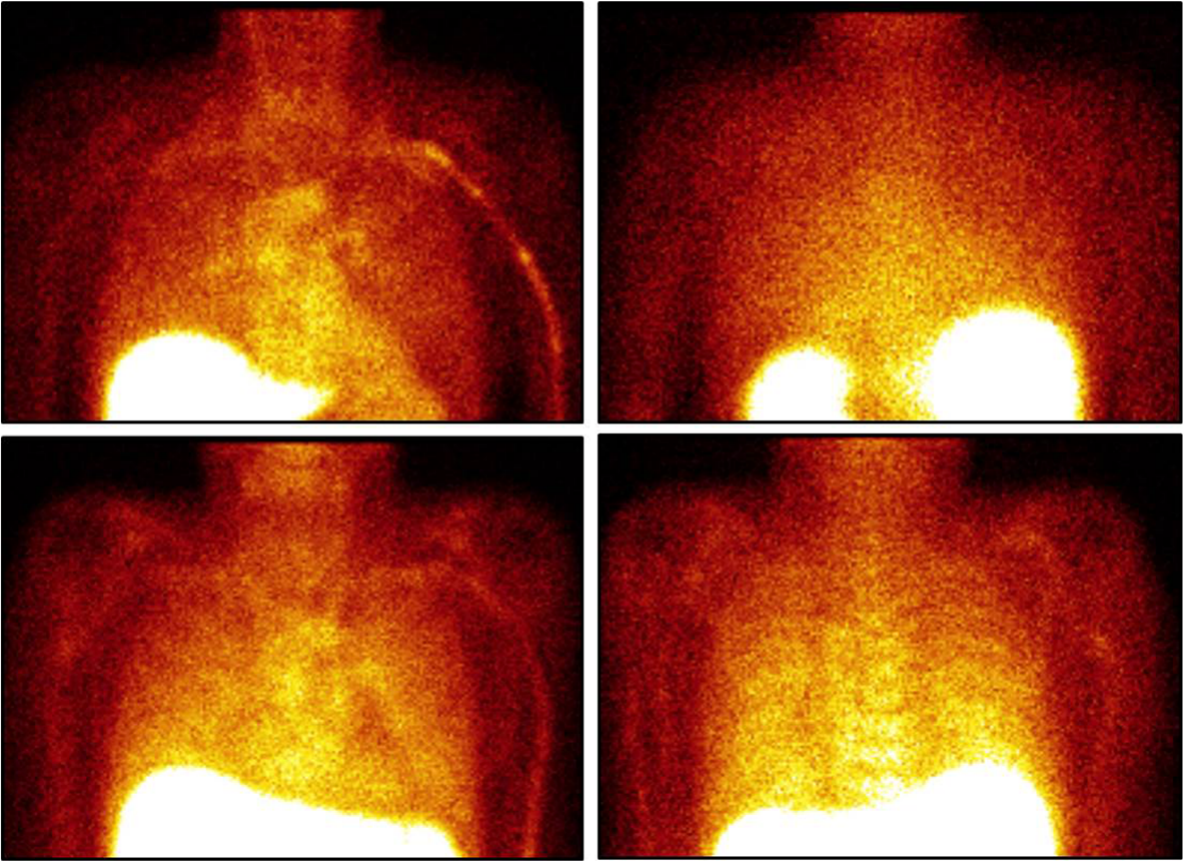
99mTc-HYNIC-IL-2 scintigraphy. Upper row images: anterior (left) and posterior (right) static view of a patient without rejection, showing intense uptake in the liver and moderate uptake in the mediastinum/blood pool. Lower row images: anterior (left) and posterior (right) static view of a patient with rejection, showing intense uptake in the liver, moderate uptake in mediastinum/blood pool, and increased uptake in the basal and posterior parts of the lungs (uptake equal to mediastinum)

## Discussion

In this proof-of-concept study, despite the low number of patients, we show that ^99m^Tc-HYNIC-IL-2 scintigraphy is able to detect acute rejection in lung transplant recipients shortly after transplantation (Fig. 1). Maybe even more important, in future, it may provide a tool to avoid transbronchial biopsies when the imaging is negative or to improve the yield of these biopsies when positive. One of the benefits of imaging acute rejection with radiolabelled IL-2 is its non-invasive nature. TBBs, which are usually performed under general anaesthesia, are invasive and may lead to complications [1]. Furthermore, imaging with radiolabelled IL-2 can assess the entire lung, whereas TBBs are taken at random throughout the lung. This may lead to sampling errors.

The one patient with acute rejection in the TBB who had a negative scan showed only minimal rejection in the TBB (grade A1). On the other hand, the 2 patients with both a positive scan and acute rejection in the TBB showed mild to moderate acute rejection (grade 2 and 3). Therefore, the sensitivity of this SPECT technique may be too low to detect minimal rejection.

Since T cells with increased IL-2R expression are also upregulated in patients with viral infections [11], we advise to exclude patients with a possible viral infection before performing radiolabelled IL-2 imaging, to increase specificity. In our study, the patients were clinically assessed prior to the scan for viral infections and showed no signs of viral infection.

In this study, we used ^99m^Tc-HYNIC-IL-2, a SPECT radiopharmaceutical. Recently, we developed a new IL-2 tracer labelled with fluorine-18 (^18^F), suitable for imaging with positron emission tomography/computed tomography (PET/CT) [12]. The use of PET/CT imaging improves image resolution and allows for the absolute quantification of IL-2 uptake in lung regions, most likely increasing sensitivity.

Our study suggests that non-invasive imaging with radiolabelled IL-2 can be a promising new tool in the detection and perhaps also the exclusion of acute rejection in patients after lung transplant. Further studies with larger patient populations are needed to determine the value of radiolabelled IL-2 imaging, preferably with the PET tracer. Possible applications of IL-2 imaging may be (1) in addition to TBB, to guide TBB to possible sites of rejection, or (2) to possibly avoid TBB in case of negative imaging.

## Conclusion

In conclusion, in this proof-of-concept study, ^99m^Tc-HYNIC-IL-2 scintigraphy proved to be a good technique to detect grades 2 and 3 acute rejection in a small sample population of patients after lung transplantation. Larger studies are necessary to really show the added value of this non-invasive specific imaging technique over TBB. Alternatively, imaging with the PET tracer ^18^F-IL-2 may be useful for this purpose.

## Abbreviations

CRP: C-reactive protein
ESR: Erythrocyte sedimentation rate
HR-CT: High-resolution computed tomography
IL-2: Interleukin-2
IL-2R: Interleukin-2 receptor
ISHLT: International Society for Heart and Lung Transplantation
NPV: Negative predictive value
PET: Positron emission tomography
PPV: Positive predictive value
SPECT: Single-photon emission computed tomography
T/B: Target-to-background
TBB: Transbronchial biopsy
